# Personality as a Possible Intervention Target to Prevent Traumatic Events in Adolescence

**DOI:** 10.3390/bs12040090

**Published:** 2022-03-25

**Authors:** Lucinda Grummitt, Emma Barrett, Erin V. Kelly, Lexine Stapinski, Nicola Newton

**Affiliations:** The Matilda Centre for Research in Mental Health and Substance Use, The University of Sydney, Sydney, NSW 2006, Australia; emma.barrett@sydney.edu.au (E.B.); erin.k@sydney.edu.au (E.V.K.); lexine.stapinski@sydney.edu.au (L.S.); nicola.newton@sydney.edu.au (N.N.)

**Keywords:** personality, trauma, adolescence, prevention

## Abstract

Traumatic events (severe injury, violence, threatened death) are commonly experienced by children. Such events are associated with a dose-response increasing risk of subsequent substance use, mental illness, chronic disease, and premature mortality. Preventing the accumulation of traumatic events is thus an urgent public health priority. Substance use risk personality profiles (impulsivity, sensation seeking, hopelessness, and anxiety sensitivity) may be an important target for preventing trauma exposure, given associations between these personality traits and risky behaviour, substance misuse, and injuries across adolescence. The current study aimed to investigate associations between personality at age 13 and the number of traumatic events experienced by age 18. It also examined associations between traumas before age 13 and personality at age 13. Participants were the control group of a cluster-randomised controlled trial examining prevention of adolescent alcohol misuse. Baseline data were collected at ages 12–13 (2012). Participants were followed-up at ages 18–19 (2017–2018). Personality profiles of hopelessness, anxiety sensitivity, impulsivity, and sensation seeking were measured at baseline using the Substance Use Risk Profile Scale. Traumatic events and age of exposure were measured at age 18–19 using the Life Events Checklist for DSM-5. Mixed-effect regression was conducted on 287 participants in Stata 17, controlling for sex. High scores on hopelessness, impulsivity, and sensation seeking at age 13 were associated with a greater number of traumatic events by age 18. Impulsivity and sensation seeking predicted the number of new traumatic events from age 13 to 18. Prior trauma exposure was associated with high hopelessness at age 13. Adolescents exhibiting high impulsivity or sensation seeking may be at greater risk of experiencing traumatic events. Additionally, early trauma exposure may contribute to the development of a hopelessness personality trait.

## 1. Introduction

Approximately one third of children are estimated to experience a traumatic event before they reach adulthood [1]. Traumatic events are defined by The Diagnostic and Statistical Manual of Mental Disorders 5 (DSM-5) as involving severe injury or illness, physical or sexual violence, or threatened death [2]. The prevalence of trauma during childhood is alarming, given documented associations with physical and psychological harms, both acutely and in the long term. Childhood traumatic events have been linked to an increased risk of subsequent substance use, mental illness, chronic disease, suicide, and premature mortality [3,4,5,6]. The disability burden caused by trauma exposure incurs an estimated annual cost of $24 billion in Australia alone [7]. The prevention of trauma exposure is thus a critical public health priority.

For individuals who do experience trauma, the majority are exposed to multiple traumatic events [8]. Epidemiological evidence confirms that trauma exposures are not randomly distributed across the population, but rather are strongly predicted by previous trauma exposure [8]. Moreover, multiple trauma types co-occur in identifiable clusters. For example, person-centred techniques, such as latent class analysis, find the majority of individuals within a sample can be classified into a low exposure class, with a minority of the sample belonging to classes experiencing a high number of co-occurring trauma types [9,10,11,12]. Importantly, a large literature has demonstrated that the risk for physical and psychological harm increases in a dose-response pattern with the number of traumatic events experienced [3,5], highlighting the urgency of intervening to prevent the accumulation of traumatic events [13].

Thus, understanding risk factors for experiencing traumatic events is critical to prevent exposure. While the bulk of research has focused on the consequences of trauma exposure, less attention has been given to identifying antecedents. While the existing research is invaluable, preventing exposure carries a huge individual, economic, and societal benefit. Research examining who may be at greater risk for experiencing traumatic events has identified risk factors operating at individual, relationship, community, and societal levels of influence [14]. These include a number of sociodemographic and structural determinants of trauma exposure, such as socioeconomic status, race/ethnicity, age, gender, marital status, neighbourhood characteristics, and sociopolitical factors, such as firearm policies [8,14]. It is crucial to address these social determinants of trauma exposure. Yet, it is also important to examine modifiable individual risk factors for trauma exposure that can be translated into interventions and resource individuals who may exhibit risk factors.

In this respect, personality is an important area for consideration. Personality refers to a persistent, underlying pattern of thoughts, emotions, and behaviours that are broadly categorised into two domains of inhibited or disinhibited personality. Research from the substance use field has identified two personality traits from each domain that are associated with increased risk of substance misuse and comorbid mental disorders [15]. This is referred to as the four-factor model of vulnerability [15]. Along the inhibited domain are the traits of hopelessness, characterised by low mood and negative feelings about the self, others, and the future, and anxiety sensitivity, characterised by a fear of anxiety-related physical sensations, such as an abnormal heartbeat or nausea. Along the disinhibited domain are the traits of impulsivity, characterised by difficulties in response inhibition, planning, and propensity toward antisocial and aggressive behaviours, and sensation-seeking, characterised by an elevated need for stimulation and an intolerance of boredom. While the four-factor model of vulnerability has been predominately examined in the context of substance use, preliminary evidence suggests these personality traits may be important for primary prevention of trauma, as well as prevention of harmful mental health and substance use consequences that may arise following trauma exposure.

### Personality and Traumatic Events

Personality develops through complex interactions between temperament (an inherited individual difference in reactivity and self-regulation), social processes, and environmental factors [16]. Differences in temperament begin to emerge in infancy, with personality developing from early childhood and solidifying across adolescence and early adulthood [16,17,18]. Both personality development and trauma exposure may be occurring early in development, resulting in reciprocal and ongoing relationships between the two. On the one hand, environmental factors, such as trauma exposure in childhood and adolescence, can impact the development of personality traits, with empirical evidence finding particularly elevated scores on measures of neurotic traits, depression, introversion, and antisocial behaviours/deviancy among children experiencing trauma before the age of five compared to after this age [19]. On the other hand, adolescents with certain personality traits may be at greater risk of experiencing traumatic events compared to peers without these traits, with empirical evidence that children exhibiting problems related to disinhibition, such as conduct disorder and oppositional defiant disorder, had an elevated risk for experiencing later interpersonal violence and other traumatic events [20]. There are also likely recursive associations whereby exposure to trauma results in personality factors that in turn may increase the likelihood of subsequent traumatic exposures [21]. Aggressive or disinhibited behaviours arising from trauma exposure may increase the likelihood of involvement in high-risk behaviours or elicit violence from others [22]. Moreover, the aforementioned substance use risk personality profiles of hopelessness, anxiety sensitivity, impulsivity, and sensation seeking may arise through trauma exposure early in life and increase the risk for traumatic events through heavy alcohol and drug consumption [23,24].

These latter pathways between personality factors and traumatic events may be more relevant for preventing exposure to traumatic events. Greater impulsivity and sensation seeking may lead individuals to engage in more risk-taking behaviours or be less conscious of the consequences of behaviours, leading to more accidents and injuries. Empirical evidence supports this notion, with a prospective study showing disinhibited traits in childhood predict traumatic brain injuries in adulthood [25]. Moreover, deficits in executive function were found to be a risk factor for exposure to interpersonal violence [26]. Those high in impulsivity have been found to endorse more traumatic events, such as physical and sexual assault [27], and impulsivity was found to be associated with lower perception of risk and greater risk-taking behaviours in adolescents [28]. Both impulsivity and sensation seeking are positively associated with risky driving practices and motor vehicle accidents [29,30]. Additionally, children exhibiting disorders related to disinhibition were found to be at elevated risk for subsequent interpersonal violence and other traumatic events [20]. However, much of the evidence is based on cross-sectional designs, which fail to account for possible recursive relationships between trauma and personality and are unable to confirm whether personality may be a way to identify those at greater risk for traumatic exposures. While relatively understudied in this context, hopelessness and anxiety sensitivity are associated with more coping motives for substance use and experiencing greater reward from the tension reduction and anxiolytic properties of substances, culminating in more risky use [15]. This may increase the possibility of experiencing traumatic events, with injury rates higher in young people who engage in hazardous drinking [24]. This is not to say that the individual is to blame for exposure to traumatic events, but rather begs the question of whether addressing emotions and behaviours associated with these personality factors could reduce experiences of traumatic events.

The utility of personality as an intervention target has been demonstrated in the field of substance misuse. For example, the Preventure program identifies adolescents scoring highly on any of the substance use risk personality profiles and delivers a personality-specific intervention to these adolescents in small groups [31]. The intervention encourages young people to understand how their personality influences their thoughts and actions and helps them identify ways of coping with these emotional and behavioural reactions that involve less risk than engagement in heavy substance use. It seems plausible that a similar approach could reduce exposure to traumatic events by promoting adaptive emotional and behavioural regulatory strategies that help to divert adolescents away from risky behaviours and substance use to cope with personality-specific reactions.

Thus, personality appears as a possible intervention target to reduce exposure to traumatic events. However, more research is needed to establish the evidence base for associations between personality traits and a range of traumatic events. Evidence as to whether, and which, personality traits can be used to predict future trauma exposure is needed to inform possible prevention efforts. Moreover, cross-sectional associations used in prior literature are unable to shed light on the recursive relationships between personality and trauma. Here, longitudinal research with adolescent samples is crucial, as it is during this period that individuals begin to engage in more independent behaviours outside of parental supervision, which may increase the potential for involvement in risky behaviours and subsequent traumatic events. Prevention efforts delivered prior to these occurrences have the greatest potential to produce traumatic events and resulting harms.

The current study aimed to address these gaps by exploring the longitudinal associations between personality traits and exposure to traumatic events across adolescence. Specifically, the current study aimed to explore the following research questions:

- Is personality at age 13 associated with the number of traumatic events experienced before age 18?
- Does personality at age 13 predict the number of new traumas experienced across adolescence?
- Do the number of trauma types experienced in early childhood predict personality trait expression at age 13?

While the current study was exploratory, we hypothesised that:

- Significant associations would be observed between traumatic events occurring from 0 to 18 years and the personality traits of hopelessness, anxiety sensitivity, impulsivity, and sensation seeking, measured at age 13.
- Impulsivity and sensation seeking would predict a greater number of new traumatic events across adolescence.
- Traumatic experiences prior to age 13 would predict a greater likelihood of expressing a personality trait of hopelessness, anxiety sensitivity, and impulsivity at age 13.

## 2. Materials and Methods

### 2.1. Sample and Design

The current sample was drawn from the Climate and Preventure (CAP) Study, a cluster-randomised controlled trial (RCT) examining the efficacy of universal and selective school-based programs in preventing adolescent alcohol misuse (trial registration ACTRN12612000026820). Two study protocols describing the original study and long-term follow ups are published elsewhere [32,33]. The current analytic sample is restricted to the control group only, to remove the possibility for any intervention effect on experiencing traumatic events.

A total of 190 schools in New South Wales and Victoria, Australia, were selected at random from a list of all public and private schools. Twenty-six schools agreed to participate, of which 7 schools were randomly allocated to the control group. Only students who received parental consent and who consented themselves to participate in the study were invited to complete student surveys.

Baseline data were collected in 2012, when participants were in Grade 8 (approximately 12–13 years of age) (Time 1). Participants were followed-up initially for three years across four survey occasions, completing surveys at six months post-baseline, then annually for three years (Time 2–5). Five to six years post-baseline (2017–2018), when participants were approximately 18–19 years of age, they completed a long-term follow-up survey (Time 6). Data for the current study are taken from Time 1 and Time 6, as relevant measures were assessed at these time points. All data was collected through anonymous self-report surveys, which took 30–40 min to complete. At each survey occasion, participants entered their unique code, which was created at baseline, allowing the participants’ responses to be linked at each survey wave but maintaining participant anonymity. Researchers were aware of these unique codes but not the identifiable information corresponding to that individual.

### 2.2. Ethical Approval

All procedures were approved by the Human Research Ethics Committees of the University of New South Wales, the University of Sydney, the Sydney Catholic Education Office, and the New South Wales Department of Education and Training.

### 2.3. Measures

Demographics (age and sex) were measured at baseline.

Personality was measured at baseline (roughly age 13) using the Substance Use Risk Profile Scale (SURPS), the screening tool used to identify students for inclusion in the selective Preventure program [33,34]. The SURPS comprises 23 items measuring the four personality risk factors for substance use problems: hopelessness, anxiety sensitivity, impulsivity, and sensation seeking. It has been validated among Australian adolescents [35], and personality measured with the SURPS shows stability across adolescence [36,37,38]. As per the Preventure scoring procedure, students scoring more than one standard deviation above the population mean on any of the four personality traits were coded as being high in the trait from which they differed most from the mean. Students who were not allocated to any high-risk personality groups were considered as having a low-risk personality type. A total of 332 students were coded as low-risk, 41 as high in hopelessness, 68 as high in anxiety sensitivity, 35 as high in impulsivity, and 51 high in sensation seeking. Thus, 37% of the sample were allocated to a high-risk personality group.

Exposure to traumatic events was measured using the Life Events Checklist for DSM-5 (LEC-5) at Time 6. This scale asks about lifetime exposure to 16 potentially traumatic events based on the conceptualisation of trauma in the DSM-5. The LEC-5 also asks whether the participant experienced any other very distressing event, or whether the participant witnessed any of the events happening to someone else. The number of different traumatic events experienced was summed for each participant to create a total exposure score. In the current study, the number of traumatic events experienced ranged from 0 to 9, with a mean of 1.24 (SD = 1.72). In addition, for each traumatic event endorsed, participants reported the age at which they first experienced that event. If the participant reported experiencing an event before the age they were at baseline (on average, age 13), they were coded as having experienced an event prior to baseline. The number of different traumatic events experienced prior to baseline was summed, giving each participant a total score of the number of trauma types experienced before baseline. This enabled an examination of recursive associations between personality and trauma, that is, associations between exposure to trauma and subsequent personality measurement, as well as between personality measurement and subsequent trauma exposure. The number of traumas experienced before baseline ranged from 0 to 6 among those who experienced any trauma, with a mean of 0.86 (SD = 1.12).

### 2.4. Analysis

Descriptive and attrition analyses were conducted in SPSS version 28. Associations between personality and traumatic events were conducted in Stata 17 using mixed effect linear (Research Questions 1 and 2) and logistic (Research Question 3) regression models, to account for clustering at the school level. Univariate models were run to test whether there were interactions between the personality groups and sex. No significant interactions were found; thus, sex was included as a covariate only in subsequent multivariate models. The suitability of the data for multiple regression was assessed and deemed acceptable, with all values of tolerance above 0.87 and all levels of variance inflation factor below 1.15. To address Research Question 1, personality groups at baseline were entered as predictors with low-risk personality as the reference group, sex was entered as a covariate, and the total number of traumatic events experienced was the outcome variable. To address Research Question 2, the same model was run, but this time controlling for the number of traumatic events experienced before baseline, to test whether personality predicted the number of new traumatic events experienced over adolescence. To address Research Question 3, univariate logistic regression models were run with each personality group as the outcome variable. The number of traumas before baseline was entered as a predictor, and sex was entered as a covariate.

## 3. Results

At baseline, 527 students completed the survey. Of these, 298 students were present at the 6-year follow-up occasion (57% of baseline). The analytical sample consists of 287 participants who had complete data on the variables of interest. The analytical sample was compared to the baseline sample on age, sex, and personality. Only sex was a significant predictor of missingness, with those retained in the sample more likely to be female (OR = 1.78, 95% CI 1.22–2.59). Demographic characteristics are presented in Table 1.

Table 2 presents the exposure to traumatic events of the sample. As shown, traumatic events were more prevalent among the high-risk personality groups (64% of the high-risk personality group experienced any trauma, compared to 43.9% of the low-risk personality group). Both the impulsivity and sensation seeking groups showed the highest prevalence of traumatic events (66.7% experienced any traumatic event). Looking at pre-adolescent trauma, both the hopelessness and sensation-seeking groups showed the highest prevalence of traumatic events.

Table 3 presents the results for Research Question 1. As shown, the high-risk groups of hopelessness (ß = 1.16, SE = 0.40), impulsivity (ß = 1.21, SE = 0.45), and sensation seeking (ß = 0.94, SE = 0.35) were associated with experiencing a greater number of trauma types by Time 6, compared to the low-risk group. Those in the anxiety sensitivity group were not significantly different to the low-risk group on the number of trauma types experienced (ß = 0.14, SE = 0.29).

Results of Research Question 2 are presented in Table 4. As shown, when controlling for prior traumatic events, a greater number of subsequent traumatic events experienced across adolescence was predicted by impulsivity (ß = 1.16, SE = 0.32) and sensation seeking (ß = 0.59, SE = 0.25). Neither hopelessness (ß = 0.26, SE = 0.29) nor anxiety sensitivity (ß = −0.07, SE = 0.21) were associated with the number of subsequent trauma experiences.

For Research Question 3, the number of traumatic events before baseline only predicted hopelessness, such that a greater number of pre-baseline traumatic events increased the odds of scoring as high-risk on hopelessness compared to low-risk personality (OR = 1.68, 95% CI 1.15–2.45). The number of pre-baseline traumatic events was not significantly associated with any of the other high risk personality types (anxiety sensitivity: OR = 1.23, 95% CI 0.83–1.84; impulsivity: OR = 1.02, 95% CI 0.54–1.94; sensation seeking: OR = 1.37, 95% CI 0.89–2.11).

## 4. Discussion

The current study found high rates of exposure to traumatic events among Australian youth, with half of participants reporting exposure to at least one traumatic event by age 18. This was markedly pronounced among youth identified as high-risk for substance misuse, among whom 64% reported exposure to any traumatic event by adulthood. This highlights the importance of understanding antecedents of trauma exposure to inform prevention efforts. In partial support of our first hypothesis, hopelessness, impulsivity, and sensation seeking were all positively associated with experiencing a greater number of traumatic events across adolescence; however, anxiety sensitivity was not associated with exposure to traumatic events. In support of our second hypothesis, both impulsivity and sensation seeking predicted new traumatic events across adolescence. Finally, our third hypothesis was partially supported, with the number of traumas experienced in early childhood predicted high-risk scores on hopelessness, but no other personality trait.

Results of the current study suggest that students scoring highly on impulsivity or sensation seeking early in adolescence may be at greater risk of experiencing traumatic events across adolescence. These results support and extend previous literature that individuals high in disinhibited traits may engage in greater risk-taking behaviours and as a result have greater exposure to traumatic events [25,29,30]. This study adds to the literature by demonstrating that early adolescent measurement of impulsivity and sensation seeking can be used to identify students at greater risk of traumatic exposures, hopefully enabling the provision of interventions to prevent these exposures. However, more evidence is needed to determine the mechanisms that explain these observed associations between personality traits and exposure to traumatic events.

Certain personality-specific cognitions and behaviours are plausible mechanisms linking impulsivity and sensation seeking to trauma exposure and could provide intervention targets. For sensation seekers, these may involve a tendency toward greater positive expectancies from risky behaviours combined with a lower perception of risk. Empirical evidence has shown that adolescents high in sensation seeking rate a range of activities across social, health, recreational, and ethical domains as less risky compared to those low in sensation seeking and that higher adolescent sensation seeking predicts greater ratings of benefit relative to cost for these risky behaviours [39]. Both of these factors are associated with a greater likelihood of engaging in risky behaviours and mediated the relationship between sensation seeking and engaging in risk-taking behaviours, accounting for 46% of the variance [39]. Similarly, in a cross-sectional study of adolescents, positive and negative alcohol expectancies mediated the association between sensation seeking and alcohol use [40]. These may be mechanisms linking sensation seeking to traumatic events as well as alcohol use, as stimulating and exciting experiences are highly valued by sensation seekers, who may anticipate greater benefits and pleasure from risky behaviours and may underestimate the possible negative consequences compared to those lower in sensation seeking. Challenging positive expectancies of high-risk behaviours and the promotion of alternate, less risky activities that fulfil the needs of sensation seekers may be important components of an intervention tailored to these adolescents. Psychoeducation of how their personality style may lead to greater risk of traumatic events and ways to mitigate this may also be important for these adolescents. In addition, while important for all adolescents, parental monitoring may be particularly important for those high in sensation seeking, with cross-sectional and longitudinal evidence of a buffering effect of parental monitoring on the relationship between sensation seeking and adolescent delinquency (substance use, sexual risk behaviours, truancy, carrying a weapon, physical fights) [41,42].

Possible intervention targets for impulsivity may include addressing the facets of negative urgency (a tendency to act rashly when faced with strong, negative emotions), and a lack of premeditation (acting hastily without regards to consequences) [43]. Both of these components were positively associated with past-year risky driving in a sample of young adults and negative urgency was additionally positively associated with past-year driving under the influence [44], both of which could lead to serious injury or threatened death. In addition, negative urgency has been found to be associated with both reactive and proactive aggression (i.e., as a response to provocation as well as pre-planned in pursuit of a secondary goal), which may increase vulnerability to involvement in assaults or severe injury [45]. Moreover, urgency has been found to be associated with difficulties in emotion regulation [46], which in turn may increase the likelihood of engaging in risky behaviours in an attempt to reduce or distract from negative emotional states [47]. Thus, promoting more adaptive emotion regulation strategies could reduce the use of more risky behaviours to cope with negative affect, and indeed, interventions targeting emotion regulation have been shown to reduce risky behaviours including self-harm, substance misuse, and risky sexual behaviour [47]. Taken together, the literature suggests that interventions promoting adolescents’ ability to slow down, think through their behaviours, and successfully regulate their emotions, particularly in the face of negative affect, may help to reduce involvement in risky behaviours arising from negative urgency and a lack of premeditation, and accordingly reduce the likelihood for exposure to traumatic events.

It is also important to consider the role of alcohol use in contributing to the observed associations. The personality traits assessed in the current study are known risk factors for adolescent substance misuse [15], and given that alcohol consumption is associated with higher rates of accidents and injuries [24], it is plausible that the associations between personality and traumatic events observed in the current study at least partly operate through a liability to risky alcohol use. Indeed, this could explain why no associations were found between anxiety sensitivity and traumatic events. Anxiety sensitivity appears to become progressively more influential in predicting substance use across development, with associations between anxiety sensitivity and substance use less commonly observed in adolescents compared to adults [34,37,48]. Thus, compared to the other personality traits measured, those high in anxiety sensitivity may have a lower risk of engaging in high-risk situations involving alcohol use over the course of the study, possibly resulting in exposure to fewer traumatic events. However, it is unlikely alcohol is the only mechanism explaining the associations between personality and traumatic events observed in the current study. Hopelessness is associated with heavy alcohol consumption in adolescents [34], yet in the current, study hopelessness was not found to predict new traumatic events across adolescence. This suggests that while alcohol likely plays a role in the associations between personality and traumatic events found in the current study, it appears to interact with personality factors such that those high in impulsivity and sensation seeking may use alcohol in more high-risk situations compared to those high in hopelessness.

The present findings also suggest a role of traumatic exposures in contributing to the expression of hopelessness. Hopelessness is related to depression vulnerability, which is frequently observed in children exposed to trauma. Indeed, a recent meta-analysis found the odds of depression were 2.6 times higher among children and adolescents exposed to trauma compared to those non-exposed or less exposed [49]. While the current study was exploratory and unable to address the complexity and timing of personality development, such results emphasise the importance of applying a trauma-informed lens to personality research. As demonstrated in the current study, many children will have experienced trauma in early childhood. Early trauma exposure may influence the development of personality expression early in the life course, as well as mental health and substance use problems [50]. This highlights the need for early intervention efforts to mitigate the impact of trauma on children.

The current study has important implications for potential interventions to prevent trauma exposure. Personality may be a non-stigmatising way of identifying those at greater risk of accidents, assaults, or injuries, who may benefit from preventive interventions. Interventions could target personality-specific factors that may increase vulnerability to exposure to traumatic events. Indeed, there are existing evidence-based interventions that address personality-risk factors for substance use problems [51,52], which demonstrate the utility of a personality-targeted approach. For example, motivational interviewing incorporating personality-specific emotions and behaviours has been found to be more effective in reducing alcohol use among young adults presenting at the emergency department for alcohol-related injuries compared to standard motivational interviewing without a personality component [52]. Moreover, in small groups of adolescents exhibiting high scores on one of the substance use risk personality profiles, the selective Preventure intervention addresses the personality-specific motivational and cognitive pathways to substance misuse and mental health symptomatology through cognitive-behavioural strategies and motivational interviewing [31]. Multiple randomised controlled trials have revealed its efficacy in reducing substance misuse and co-occurring mental health symptoms among these high-risk adolescents [51]. These existing interventions may reduce exposure to traumatic events or could be adapted to pursue this aim, perhaps by expanding psychoeducation to include awareness that some personality styles can lead to greater risk of traumatic events and identifying ways to mitigate this. Encouragingly, Preventure involves only 2, 90-min workshops, highlighting that relatively little time and costs could have a substantial impact by preventing the negative sequelae of trauma. Future research should thus examine the efficacy of these existing programs in reducing exposure to traumatic events.

Several limitations of the current study should be noted. First, the number of traumatic events experienced before baseline was constructed from participant responses at age 18, not baseline, and thus required retrospective recall for events occurring over 5 years previously. This reporting may be subject to problems with accurate recall. Second, substantial attrition occurred over the six-year follow-up period. Attrition analyses revealed none of the high-risk trauma types predicted attrition, however it is possible that other, unmeasured factors may have biased the remaining sample. Third, data collection was completed in 2018, and thus may not capture recent trends in exposure to traumatic events. However, the study is strengthened by longitudinal data spanning adolescence and there is no reason to believe the demonstrated associations would be substantially different four years later. Moreover, the current study did not measure whether participants received therapeutic care or interventions following trauma. This may affect the demonstrated association between pre-baseline trauma exposure and hopelessness, as well as possibly reducing subsequent traumas through therapeutic intervention. Future research should incorporate measures of care following trauma exposure to probe how this may influence associations between personality and trauma exposure. Finally, the current study cannot deem whether the observed associations are causal. There are likely unmeasured confounders that partly explain these associations, which should be considered in future research. In particular, genetic or familial factors may be associated with both high-risk personality and being exposed to trauma, for example, having an impulsive parent. The current study was unable to account for these potentially confounding parental factors. Nonetheless, the current study provides preliminary evidence demonstrating associations between adolescent personality and exposure to traumatic events, that should be used to inform future research and possible preventive efforts.

## Figures and Tables

**Table 1:** Demographic and personality characteristics of the sample, N = 287.

Age	Mean, SD
Baseline	12.99, SD = 0.40
Time 6	18.44, SD = 0.52
Sex	N (%)
Females	208 (72.5)
Males	79 (27.5)
Personality	N (%)
Any high risk	100 (34.8)
Hopelessness	19 (6.6)
Anxiety Sensitivity	39 (13.6)
Impulsivity	15 (5.2)
Sensation Seeking	27 (9.4)

**Table 2:** Prevalence of traumatic events by personality.

Number of Trauma Types Experienced	Total Sample (N = 287)	Low-Risk N = 187	H N = 19	AS N = 39	IMP N = 15	SS N = 27
0	141 (49.1%)	105 (56.1%)	7 (36.8%)	15 (38.5%)	5 (33.3%)	9 (33.3%)
1	58 (20.2%)	30 (16.0%)	3 (15.8%)	15 (38.5%)	4 (26.7%)	6 (22.2%)
2	33 (11.5%)	23 (12.3%)	2 (10.5%)	3 (7.7%)	0 (0%)	5 (18.5%)
3	21 (7.3%)	13 (7.0%)	2 (10.5%)	3 (7.7%)	1 (6.7%)	2 (7.4%)
4	19 (6.6%)	11 (5.9%)	2 (10.5%)	1 (2.6%)	3 (20%)	2 (7.4%)
5	5 (1.7%)	4 (2.1%)	0 (0%)	1 (2.6%)	0 (0%)	0 (0%)
6	3 (1.0%)	1 (0.5%)	1 (5.3%)	1 (2.6%)	0 (0%)	0 (0%)
7	6 (2.1%)	0 (0%)	2 (10.5%)	0 (0%)	2 (13.3%)	2 (7.4%)
9	1 (0.3%)	0 (0%)	0 (0%)	0 (0%)	0 (0%)	1 (3.7%)
Any trauma ^a^	146 (50.9%)	82 (43.9%)	12 (63.2%)	24 (61.5%)	10 (66.7%)	18 (66.7%)
Any trauma ^a^ before baseline	73	39	7	12	4	11
	50.3% ^b^	48.1% ^b^	58.3% ^b^	50% ^b^	40% ^b^	61.1% ^b^
	25.4% ^c^	20.9% ^c^	36.8% ^c^	30.8% ^c^	26.7% ^c^	40.7% ^c^

H: hopelessness; AS: anxiety sensitivity; IMP: impulsivity; SS: sensation seeking. ^a^: Endorsed one or more trauma types; ^b^: of those who experienced any trauma; ^c^: of the total n for the column.

**Table 3:** Associations between personality groups at baseline and number of traumas experienced across adolescence.

	Coefficient (SE)	95% CI	*p* Value
H	1.160 (0.400)	0.376–1.944	0.004 **
AS	0.142 (0.293)	−0.433–0.717	0.628
IMP	1.208 (0.452)	0.323–2.093	0.007 **
SS	0.937 (0.345)	0.262–1.613	0.007 **

Multivariate models entering all personality types, controlling for sex. SE: standard error; CI: confidence interval. * *p* < 0.05; ** *p* < 0.01.

**Table 4:** Associations between personality groups at baseline and number of new traumas experienced across adolescence.

	Coefficient (SE)	95% CI	*p* Value
H	0.256 (0.289)	−0.312–0.823	0.377
AS	−0.072 (0.209)	−0.482–0.338	0.730
IMP	1.168 (0.321)	0.538–1.798	<0.001 **
SS	0.593 (0.246)	0.111–1.075	0.016 *

Multivariate models entering all personality types, controlling for sex and number of traumas experienced prior to baseline personality measurement. SE: standard error; CI: confidence interval. * *p* < 0.05; ** *p* < 0.01.

## Data Availability

Due to participant privacy and ethics requirements, data from the current study is not available to be shared.

## References

[1] Lewis S.J., Arseneault L., Caspi A., Fisher H.L., Matthews T., Moffitt T.E., Odgers C.L., Stahl D., Teng J.Y., Danese A. (2019). The epidemiology of trauma and post-traumatic stress disorder in a representative cohort of young people in England and Wales. Lancet Psychiatry.

[2] American Psychiatric Association (2013). Diagnostic and Statistical Manual of Mental Disorders.

[3] Felitti V.J., Anda R.F., Nordenberg D., Williamson D.F., Spitz A.M., Edwards V., Koss M.P., Marks J.S. (1998). Relationship of childhood abuse and household dysfunction to many of the leading causes of death in adults. The adverse childhood experiences (ace) study. Am. J. Prev. Med..

[4] Gondek D., Patalay P., Lacey R.E. (2021). Adverse childhood experiences and multiple mental health outcomes through adulthood: A prospective birth cohort study. SSM-Ment. Health.

[5] Grummitt L.R., Kreski N.T., Kim S.G., Platt J., Keyes K.M., McLaughlin K.A. (2021). Association of childhood adversity with morbidity and mortality in us adults: A systematic review. JAMA Pediatr..

[6] Petruccelli K., Davis J., Berman T. (2019). Adverse childhood experiences and associated health outcomes: A systematic review and meta-analysis. Child Abus. Negl..

[7] Kezelman C., Hossack N., Stavropoulos P., Burley P. (2015). The Cost of Unresolved Childhood Trauma and Abuse in Adults in Australia.

[8] Benjet C., Bromet E., Karam E.G., Kessler R.C., McLaughlin K.A., Ruscio A.M., Shahly V., Stein D.J., Petukhova M., Hill E. (2015). The epidemiology of traumatic event exposure worldwide: Results from the World Mental Health Survey Consortium. Psychol. Med..

[9] Barboza G.E. (2017). Latent Classes and Cumulative Impacts of Adverse Childhood Experiences. Child Maltreat..

[10] Debowska A., Willmott D., Boduszek D., Jones A.D. (2017). What do we know about child abuse and neglect patterns of co-occurrence? A systematic review of profiling studies and recommendations for future research. Child Abus. Negl..

[11] Haahr-Pedersen I., Perera C., Hyland P., Vallières F., Murphy D., Hansen M., Spitz P., Hansen P., Cloitre M. (2020). Females have more complex patterns of childhood adversity: Implications for mental, social, and emotional outcomes in adulthood. Eur. J. Psychotraumatol..

[12] Lanier P., Maguire-Jack K., Lombardi B., Frey J., Rose R.A. (2017). Adverse Childhood Experiences and Child Health Outcomes: Comparing Cumulative Risk and Latent Class Approaches. Matern. Child Health J..

[13] Hughes K., Bellis M.A., Hardcastle K.A., Sethi D., Butchart A., Mikton C., Jones L., Dunne M.P. (2017). The effect of multiple adverse childhood experiences on health: A systematic review and meta-analysis. Lancet Public Health.

[14] Magruder K.M., McLaughlin K.A., Borbon D.L.E. (2017). Trauma is a public health issue. Eur. J. Psychotraumatol..

[15] Castellanos-Ryan N., Conrod P., Verster J.C., Brady K., Galanter M., Conrod P. (2012). Personality and substance misuse evidence for a four-factor model of vulnerability. Drug Abuse and Addiction in Medical Illness: Causes, Consequences and Treatment.

[16] Xu Y., Krieg A.W., Wright J.D. (2015). Personality development and temperament. International Encyclopedia of the Social & Behavioral Sciences.

[17] Wängqvist M., Lamb M.E., Frisén A., Hwang C.P. (2015). Child and Adolescent Predictors of Personality in Early Adulthood. Child Dev..

[18] Van Dijk M.P.A., Hale Iii W.W., Hawk S.T., Meeus W., Branje S. (2020). Personality development from age 12 to 25 and its links with life transitions. Eur. J. Personal..

[19] Rutkowski K., Dembińska E., Walczewska J. (2016). Effect of trauma onset on personality traits of politically persecuted victims. BMC Psychiatry.

[20] Carliner H., Gary D., McLaughlin K.A., Keyes K.M. (2017). Trauma Exposure and Externalizing Disorders in Adolescents: Results from the National Comorbidity Survey Adolescent Supplement. J. Am. Acad. Child Adolesc. Psychiatry.

[21] Pos K., Boyette L.L., Meijer C.J., Koeter M., Krabbendam L., De Haan L., for GROUP (2016). The effect of childhood trauma and Five-Factor Model personality traits on exposure to adult life events in patients with psychotic disorders. Cogn. Neuropsychiatry.

[22] Bynion T.-M., Cloutier R., Blumenthal H., Mischel E.R., Rojas S.M., Leen-Feldner E.W. (2018). Violent interpersonal trauma predicts aggressive thoughts and behaviors towards self and others: Findings from the National Comorbidity Survey-Adolescent Supplement. Soc. Psychiatry.

[23] Grummitt L., Kelly E., Barrett E., Keyes K., Newton N. (2021). Targets for intervention to prevent substance use in young people exposed to childhood adversity: A systematic review. PLoS ONE.

[24] Lester L., Baker R., Coupland C., Orton E. (2017). Alcohol Misuse and Injury Outcomes in Young People Aged 10–24. J. Adolesc. Health.

[25] Guberman G.I., Robitaille M.-P., Larm P., Ptito A., Vitaro F., Tremblay R.E., Hodgins S. (2019). A Prospective Study of Childhood Predictors of Traumatic Brain Injuries Sustained in Adolescence and Adulthood. Can. J. Psychiatry.

[26] Fazel S., Smith E.N., Chang Z., Geddes J.R. (2018). Risk factors for interpersonal violence: An umbrella review of meta-analyses. Br. J. Psychiatry.

[27] Hautmann A.K. (2019). A Comprehensive Analysis of Impulsivity in Individuals with and without Trauma Exposure: Rochester Institute of Technology. Bachelor’s Thesis.

[28] Reniers R.L.E.P., Murphy L., Lin A., Bartolomé S.P., Wood S.J. (2016). Risk perception and risk-taking behaviour during adolescence: The influence of personality and gender. PLoS ONE.

[29] Zhang X., Qu X., Tao D., Xue H. (2018). The association between sensation seeking and driving outcomes: A systematic review and meta-analysis. Accid. Anal. Prev..

[30] Bıçaksız P., Özkan T. (2016). Impulsivity and driver behaviors, offences and accident involvement: A systematic review. Transp. Res. Part F Traffic Psychol. Behav..

[31] Castellanos N., Conrod P. (2006). Brief interventions targeting personality risk factors for adolescent substance misuse reduce depression, panic and risk-taking behaviours. J. Ment. Health.

[32] Newton N.C., Stapinski L., Slade T., Champion K.E., Barrett E., Chapman C., Smout A., Lawler S., Mather M., Castellanos-Ryan N. (2018). Pathways to prevention: Protocol for the CAP (Climate and Preventure) study to evaluate the long-term effectiveness of school-based universal, selective and combined alcohol misuse prevention into early adulthood. BMC Public Health.

[33] Newton N.C., Teesson M., Barrett E.L., Slade T., Conrod P.J. (2012). The CAP study, evaluation of integrated universal and selective prevention strategies for youth alcohol misuse: Study protocol of a cluster randomized controlled trial. BMC Psychiatry.

[34] Woicik P.A., Stewart S.H., Pihl R.O., Conrod P.J. (2009). The substance use risk profile scale: A scale measuring traits linked to reinforcement-specific substance use profiles. Addict. Behav..

[35] Newton N.C., Barrett E.L., Castellanos-Ryan N., Kelly E., Champion K.E., Stapinski L., Conrod P.J., Slade T., Nair N., Teesson M. (2015). The validity of the Substance Use Risk Profile Scale (SURPS) among Australian adolescents. Addict. Behav..

[36] Ali A., Carré A., Hassler C., Spilka S., Vanier A., Barry C., Berthoz S. (2016). Risk factors for substances use and misuse among young people in france: What can we learn from the substance use risk profile scale?. Drug Alcohol Depend..

[37] Krank M., Stewart S.H., O’Connor R., Woicik P.B., Wall A.-M., Conrod P.J. (2011). Structural, concurrent, and predictive validity of the Substance Use Risk Profile Scale in early adolescence. Addict. Behav..

[38] Mathijssen J., Rozema A., Hiemstra M., Jansen M., van Oers J. (2021). Stability of and change in substance use risk personality: Gender differences and smoking cigarettes among early adolescents. Addict. Behav. Rep..

[39] Zhang L., Zhang C., Shang L. (2016). Sensation-seeking and domain-specific risk-taking behavior among adolescents: Risk perceptions and expected benefits as mediators. Personal. Individ. Differ..

[40] Urbán R., Kökönyei G., Demetrovics Z. (2008). Alcohol outcome expectancies and drinking motives mediate the association between sensation seeking and alcohol use among adolescents. Addict. Behav..

[41] Wang B., Deveaux L., Lunn S., Dinaj-Koci V., Li X., Stanton B. (2016). The influence of sensation-seeking and parental and peer influences in early adolescence on risk involvement through middle adolescence: A structural equation modeling analysis. Youth Soc..

[42] Mann F.D., Kretsch N., Tackett J.L., Harden K.P., Tucker-Drob E.M. (2015). Person×environment interactions on adolescent delinquency: Sensation seeking, peer deviance and parental monitoring. Personal. Individ. Differ..

[43] Whiteside S.P., Lynam D.R. (2001). The Five Factor Model and impulsivity: Using a structural model of personality to understand impulsivity. Personal. Individ. Differ..

[44] Luk J., Trim R., Karyadi K.A., Curry I., Hopfer C.J., Hewitt J.K., Stallings M.C., Brown S.A., Wall T.L. (2017). Unique and interactive effects of impulsivity facets on reckless driving and driving under the influence in a high-risk young adult sample. Personal. Individ. Differ..

[45] Hecht L.K., Latzman R.D. (2015). Revealing the nuanced associations between facets of trait impulsivity and reactive and proactive aggression. Personal. Individ. Differ..

[46] Ceschi G., Billieux J., Hearn M., Fürst G., Van Der Linden M. (2014). Trauma exposure interacts with impulsivity in predicting emotion regulation and depressive mood. Eur. J. Psychotraumatol..

[47] Weiss N.H., Sullivan T.P., Tull M.T. (2015). Explicating the role of emotion dysregulation in risky behaviors: A review and synthesis of the literature with directions for future research and clinical practice. Curr. Opin. Psychol..

[48] Kelly E.V., Grummitt L., Teesson M., Newton N.C. (2019). Associations between personality and uptake of tobacco smoking: Do they differ across adolescence?. Drug Alcohol Rev..

[49] Vibhakar V., Allen L.R., Gee B., Meiser-Stedman R. (2019). A systematic review and meta-analysis on the prevalence of depression in children and adolescents after exposure to trauma. J. Affect. Disord..

[50] Schaefer J., Moffitt T.E., Arseneault L., Danese A., Fisher H.L., Houts R., Sheridan M., Wertz J., Caspi A. (2017). Adolescent Victimization and Early-Adult Psychopathology: Approaching Causal Inference Using a Longitudinal Twin Study to Rule Out Noncausal Explanations. Clin. Psychol. Sci..

[51] Conrod P.J. (2016). Personality-Targeted Interventions for Substance Use and Misuse. Curr. Addict. Rep..

[52] Hides L., Quinn C., Chan G., Cotton S., Pocuca N., Connor J.P., Witkiewitz K., Daglish M.R.C., Young R.M.D., Stoyanov S. (2020). Telephone-based motivational interviewing enhanced with individualised personality-specific coping skills training for young people with alcohol-related injuries and illnesses accessing emergency or rest/recovery services: A randomized controlled trial (QuikFix). Addiction.

